# 术前短期高强度肺康复训练对肺癌合并COPD患者围手术期并发症的影响

**DOI:** 10.3779/j.issn.1009-3419.2018.11.06

**Published:** 2018-11-20

**Authors:** 胜蓝 孟, 帆 杨, 富强 戴, 爽 陈, 朝琼 黄, 群友 谭, 会军 牛

**Affiliations:** 400042 重庆，陆军军医大学第三附属医院(野战外科研究所) The Third Affiliated Hospital of Army Medical University (Research Institute of Surgery), Chongqing 400042, China

**Keywords:** 肺肿瘤, COPD, 肺康复训练, 肺部并发症, Lung neoplasms, COPD, Lung rehabilitation, Pulmonary complications

## Abstract

**背景与目的:**

慢性阻塞性肺病(chronic obstructive pulmonary diseases, COPD)降低患者心肺功能，可导致患者围手术期风险增加。本研究拟探讨术前短期高强度肺康复训练对肺癌合并COPD患者肺功能及术后并发症的影响。

**方法:**

分析2016年6月-2016年12月101例肺癌合并COPD患者的临床资料，其中肺康复训练组43例，常规治疗组58例，比较两组患者肺功能、术后肺部并发症、住院时间等指标，同时比较肺康复训练组训练前后肺功能相关指标。

**结果:**

两组患者术前一般资料及肺功能无显著差异，康复训练组住院时间更长[(17.23±4.18) d *vs* (14.41±4.03) d]，但术后住院时间与常规治疗组无显著差异[(8.93±3.78) d *vs* (9.62±3.98) d]，两组患者术后肺部感染[8例(18.6%) *vs* 17例(29.3%)]、肺不张[1例(2.3%) *vs* 1例(1.7%)]、呼吸衰竭[1例(2.3%) *vs* 2例(3.4%)]等无显著差异。肺康复训练组训练前后FEV_1_[(2.06±0.45) L *vs* (2.15±0.45) L, *P* < 0.001]、PEF[(4.32±0.90) L/s *vs* (5.15±1.05) L/s, *P* < 0.001]、PCO_2_[(42.42±2.79) mmHg *vs* (41.58±2.98) mmHg, *P*=0.009]改善明显，按亚组分析，康复训练后中-重度COPD亚组FEV_1_[(0.16±0.05) L (8.6%) *vs* (0.06±0.05) L (2.8%)]增加值较轻度COPD亚组更明显。

**结论:**

术前短期肺康复训练可改善肺癌合并COPD患者肺功能，其中中-重度COPD患者肺功能改善更明显。

## Abstract (en)

**Background and objective:**

Chronic obstructive pulmonary disease (COPD) will reduce the cardiopulmonary function and increase perioperative risk. The aim of this study is to investigate the effect of preoperative short-term high intensity lung rehabilitation training on lung function and postoperative complications in patients with COPD who are eligible for lung cancer surgery.

**Methods:**

We analysis of 101 patients with COPD and a diagnosis of lung cancer, with 43 patients in pulmonary rehabilitation group and 58 patients in conventional group. The pulmonary function, postoperative pulmonary complications (PPCs) and length of stay (LOS) will be compared between the two groups, the lung function will be compared before and after the rehabilitation at the same time.

**Results:**

There were no significant difference between the two groups in general information, lung function before surgery, postoperative pulmonary infection [8 (18.6%) *vs* 17 (29.3%)], atelectasis [1 (2.3%) *vs* 1 (1.7%)], respiratory failure [1 (2.3%) *vs* 2 (3.4%)] and postoperative LOS [(8.93±3.78) d *vs* (9.62±3.98) d, *P* > 0.05]. In the rehabilitation group, the FEV_1_ [(2.06±0.45) L *vs* (2.15±0.45) L, *P* < 0.001] and PEF [(4.32±0.90) L/s *vs* (5.15±1.05) L/s, *P* < 0.001) were higher, and PCO_2_ [(42.42±2.79) mmHg *vs* (41.58±2.98) mmHg, *P*=0.009] was lower after rehabilitation, significantly. The increase value of FEV_1_ in moderate to severe COPD group was higher than that of the mild COPD group after the rehabilitation [(0.16±0.05) L, 8.6% *vs* (0.06±0.05) L, 2.8%, *P* < 0.001).

**Conclusion:**

The short-term highly-intensity lung rehabilitation can improve lung function in lung cancer patients with COPD, and the improvement of pulmonary function in moderate to severe COPD patients is more obviously.

肺癌是全球男性和发达国家女性因癌症死亡率最高的疾病种类，手术仍然是目前最有效的治疗手段，但由于疾病分期、基础肺功能以及合并基础疾病等影响，只有不到30%的肺癌患者能够接受手术治疗。心肺功能不全是早-中期肺癌患者不能耐受手术的最常见原因。随着人口年龄结构老龄化及环境因素影响，慢性阻塞性肺病(chronic obstructive pulmonary diseases, COPD)发病率高居不下，肺癌人群中，文献^[1]^报道COPD发病率男性可达72.8%，女性可达52.5%。COPD患者由于肺通气及换气功能下降，长期氧摄入不足导致肌肉力量下降、活动能力差、全身炎症反应，这类患者围手术期风险及术后并发症明显升高，甚至部分早期肺癌患者因基础肺功能差而丧失手术机会。经典的肺康复训练能够改善患者肺功能、活动能力，减少住院时间，降低术后并发症发生，但由于所需时间长^[2]^，患者及其医生可能顾虑肺癌病情进展而不愿选择，目前临床上仍缺乏一种成熟、广泛接受的短期术前肺康复训练方法。本研究拟针对肺癌合并COPD的患者，探索术前安全、可行且有效的短期肺康复训练方案，现将结果汇报如下。

## 1 资料与方法

### 1.1 一般资料

2016年6月-2016年12月完成肺癌合并COPD患者胸腔镜肺叶切除+纵隔淋巴结清扫共101例，按是否行术前肺康复训练分为两组：肺康复训练(preoperative pulmonary rehabilitation, PPR)组和常规治疗(conventional scheme, CS)组。各组患者按COPD严重程度分别分为两亚组：轻度COPD组、中-重度COPD组(由于重度COPD患者数量少，与中度COPD患者合并统计)，分组标准参照慢性阻塞性肺疾病(简称慢阻肺)全球倡议2017版(GOLD)^[3]^。见表 1。纳入标准：①手术方式为胸腔镜下肺叶切除+纵隔淋巴结清扫，淋巴结清扫范围：右侧必须清扫第2、3a、4、7、8、9、10、11组淋巴结，左侧必须清扫第5、6、7、8、9、10、11组淋巴结；②术后病理活检为非小细胞肺癌；③术前符合慢性阻塞性肺病标准；④术前无心肌梗塞史，无心律失常、心脏手术史等。排除标准：①术后病理结果为小细胞肺癌；②手术方式为全肺切除、机器人肺叶切除、开放手术、辅助小切口手术、楔形切除、肺段切除或未行淋巴清扫而仅行采样；③远处孤立性转移行姑息性切除；④双侧病变行同期或分期手术；⑤合并其他疾病并同期手术，或距前次手术时间不足2周。COPD患者气流受限严重程度分级标准：轻度(GOLD 1)：FEV_1_/FVC < 70%，且FEV_1_%预计值≥80%；中度(GOLD 2)：FEV_1_/FVC < 70%，且50%≤FEV_1_%预计值< 80%；重度(GOLD 3)：FEV_1_/FVC < 70%，且30%≤FEV_1_%预计值< 50%；极重度(GOLD 4)：FEV_1_/FVC < 70%，且FEV_1_%预计值< 30%。

**1:** 患者一般资料 Baseline of the patients

	PPR group (*n*=43)	CS group (*n*=58)	Test value	*P*
Age (Mean±SD, yr)	58.9±8.9	61.1±9.1	*t*=0.308	0.580
Gender			*χ*^2^=0.117	0.733
Male	26	37		
Female	17	21		
Pulmonary function				
FEV_1_ (L)	2.06±0.45	2.16±0.47	*t*=1.074	0.285
FVC (L)	3.16±0.68	3.27±0.68	*t*=0.830	0.409
PEF (L/s)	4.32±0.90	4.51±0.99	*t*=0.946	0.346
MVV (L/min)	86.69±16.56	90.59±15.60	*t*=1.210	0.229
Severity of COPD			*χ*^2^=0.117	0.494
Mild	30	44		
Moderate to severe	13	14		
Resection range			*χ*^2^=3.173	0.787
RUL	18	27		
RML	2	1		
RLL	8	9		
RU-ML	0	1		
RM-LL	1	0		
LUL	7	11		
LLL	7	9		
Pathology			*χ*^2^=0.218	0.897
SC	16	19		
AC	25	36		
Other types	2	3		
Pathological stage			*χ*^2^=1.576	0.455
Stage Ⅰ	21	32		
Stage Ⅱ	11	17		
Stage Ⅲ	11	9		
Stage Ⅳ	0	0		
PPR: preoperative pulmonary rehabilitation; CS: conventional scheme; FEV_1_: forced expiratory volume in one second; FVC: forced vital capacity; MVV: maximum ventilatory volume; PEF: peak expiratory flow; COPD: chronic obstructive pulmonary disease; RUL: right upper lobe; RML: right middle lobe; RLL: right lower lobe; RU-ML: right upper middle lobe; RM-LL: right middle lower lobe; LUL: left upper lobe; LLL: left lower lobe; SC: squamous carcinoma; AC: adenocarcinoma.				

### 1.2 肺康复训练方案

术前肺康复训练时间共7 d-10 d，分药物康复及物理康复两方面。

#### 1.2.1 药物康复方案

药物康复方案与物理康复方案同期进行，具体如下：①祛痰治疗：盐酸氨溴索注射液30 mg静滴*bid*；②糖皮质激素：布地奈德1 mg高频雾化吸入*bid*；③支气管扩张剂：特布他林5 mg高频雾化吸入*bid*，或沙丁胺醇5 mg高频雾化吸入*bid*；④抗感染治疗：术前明确合并感染者给予抗感染治疗，抗感染治疗方案根据微生物药敏试验、临床药学室会诊或呼吸科会诊后制订。

#### 1.2.2 物理康复方案

物理康复方案以呼吸肌训练及心肺功能训练为主，具体如下：①呼吸训练器训练：采用三球式呼吸训练器(MSC UTRI 4311/三球式呼吸训练器)，患者取端坐位，平静呼气后含住训练器吸气嘴，用力吸气并尽量维持训练器三球悬浮于上端，吸气结束后移开吸气嘴，缩唇呼气，重复练习，每15次-20次为一组，每2 h一组，每日6组-8组；②腹式呼吸训练：患者取平卧位，经鼻吸气至最大肺容量后屏气2 s-3 s，经口缓慢缩唇呼气，吸气时腹部外凸，呼气时腹部内凹，吸呼比1:2，每组20次，每日2组；③登楼梯训练：患者在医护人员陪同下，连续登楼梯运动，运动时采用缩唇呼吸法，如出现明显心慌、气促、呼吸困难等可短暂休息，并尽快恢复运动，每日20 min-30 min。

### 1.3 观察指标

#### 1.3.1 心肺功能指标

##### 1.3.1.1 动脉血气分析

患者静息30 min以上，未吸氧状态下抽取桡动脉或足背动脉血，以美国ABBOTT公司i-STAT2000血液分析仪行动脉血气分析检查，并记录pH、氧分压(partial pressure of oxygen, PO_2_)、二氧化碳分压(partial pressure of carbon dioxide, PCO_2_)、动脉血氧饱和度(arterial oxygen saturation, SaO_2_)等指标。常规治疗组术前检查动脉血气，如多次检查则采用术前最后一次检查结果；康复训练组入院时(康复训练前)、术前(康复训练后)均检查动脉血气，如多次检查则分别采用康复训练前、术前最后一次检查结果。

##### 1.3.1.2 肺功能

采用比利时麦迪公司medi-soft Hyp Air pro肺功能仪检查患者肺功能。检查时患者取端坐位或站立位，配合检查者指令完成检查，检查结束后至少记录FEV_1_、FEV_1_%预计值、FVC、PEF、MVV等数值。常规治疗组术前检查肺功能，如多次检查则采用术前最后一次检查结果；康复训练组入院时(康复训练前)、术前(康复训练后)均检查肺功能，如多次检查则分别采用康复训练前、术前最后一次检查结果。

#### 1.3.2 术后延迟气管拔管

患者术后当日或第二日复查床旁胸片、动脉血气后常规脱机、拔除气管插管，术后呼吸机使用时间超过24 h定义为延迟气管拔管，并记录拔管时间。

#### 1.3.3 术后肺部感染及肺不张

术后肺部感染是指术后胸部平片检查发现新出现的片状影或浸润影，并同时至少满足以下1条：①白细胞(white blood cell, WBC)总数 > 12×10^9^/L或 < 4×10^9^/L；②体温 > 38.5 ℃；③新出现的浓痰或痰培养/纤支镜灌洗物培养发现致病菌；④抗生素使用时间延长。

术后肺不张：胸部X线/CT发现肺部均匀性密度增高影，多呈现三角形，尖端指向肺门；或纤支镜检查发现支气管内痰栓、血栓等完全阻塞管腔。

### 1.4 统计学方法

应用SPSS 22.0进行数据分析，计数资料采用卡方检验，计量资料采用独立样本*t*检验或配对*t*检验分析。以*P* < 0.05为差异有统计学意义。

## 2 结果

### 2.1 基本资料

PPR组共纳入患者43例，平均年龄(58.9±8.9)岁，其中男性26例，女性17例，按照COPD诊断及分级标准，轻度COPD 30例，中-重度COPD 13例；CS组共纳入病例58例，平均年龄(61.1±9.1)岁，其中男性37例，女性21例，轻度COPD 44例，中-重度COPD 14例。两组患者一般资料比较无统计学差异(*P* > 0.05)，两组患者入院时肺功能、手术切除部位、术后病理类型、临床分期等结果比较无统计学差异(*P* > 0.05)。结果见表 1。

### 2.2 肺康复训练前后心肺功能

PPR组患者完成康复训练后，术前再次复查肺功能、动脉血气等指标。其中康复训练前后患者FVC、MVV、pH、PO_2_、SaO_2_等结果差异无统计学意义(*P* > 0.05)；FEV_1_[(2.06±0.45) L *vs* (2.15±0.45) L)]、PEF[(4.32±0.90) L/s *vs* (5.15±1.05) L/s]、PCO_2_[(2.42±2.79) mmHg *vs* (41.58±2.98) mmHg)]康复训练前后差异有统计学意义(*P* < 0.05)，结果见表 2。按患者COPD分级不同，轻度COPD亚组患者FEV_1_[(2.06±0.45) L *vs* (2.15±0.45) L]，平均增加值[(0.06±0.05) L (2.8%)]、PEF[(4.53±0.75) L/s *vs* (5.34±0.91) L/s]，平均增加值[(0.81±0.23) L/s (17.9%)]康复训练后显著增加(*P* < 0.05)，其余指标无统计学差异；中-重度COPD亚组康复训练前后肺功能FEV_1_[(1.85±0.42) L *vs* (2.01±0.41) L]，平均增加值[(0.16±0.05) L (8.6%)]、PEF[(3.85±1.06) L/s *vs* (4.72±1.26) L/s]，平均增加值[(0.87±0.21) L/s (22.6%)]、PCO2[(41.85±3.24) mmHg *vs* (39.62±3.88) mmHg]差异明显(*P* < 0.05)，其余指标无明显差异(*P* > 0.05)，且中-重度COPD亚组康复训练前后肺功能改善值较轻度COPD亚组更明显，结果见表 2、表 3。

**2:** PPR组心肺功能情况（Mean±SD） Pulmonary function of PPR group (Mean±SD)

	Pre-rehabilitation	Post-rehabilitation	*t*	*P*
FEV_1_ (L)	2.06±0.45	2.15±0.45	8.729	0.000
FVC (L)	3.16±0.68	3.18±0.67	0.702	0.487
PEF (L/s)	4.32±0.90	5.15±1.05	23.690	0.000
MVV (L/min)	86.69±16.56	88.16±17.83	1.636	0.109
ABG				
pH	7.41±0.04	7.42±0.03	1.652	0.106
PO_2_ (mmHg)	73.91±6.46	74.14±5.73	0.848	0.401
PCO_2_ (mmHg)	42. 42±2.79	41.58±2.98	2.746	0.009
SaO_2_ (%)	93.77±2.81	93.67±2.53	0.599	0.553
Mild COPD				
FEV_1_ (L)	2.16±0.44	2.22±0.47	6.822	0.000
FVC (L)	3.25±0.66	3.25±0.65	0.144	0.877
PEF (L/s)	4.53±0.75	5.34±0.91	18.960	0.000
MVV (L/min)	88.70±15.25	89.60±16.27	0.867	0.393
ABG				
pH	7.40±0.03	7.41±0.03	1.668	0.106
PO_2_ (mmHg)	76.30±5.27	76.17±4.72	0.441	0.662
PCO_2_ (mmHg)	42.67±2.59	42.43±2.03	0.793	0.434
SaO_2_ (%)	94.90±2.29	94.63±2.11	1.393	0.174
Moderate to severe COPD				
FEV_1_ (L)	1.85±0.42	2.01±0.41	11.954	0.000
FVC (L)	2.97±0.69	3.02±0.66	1.124	0.283
PEF (L/s)	3.85±1.06	4.72±1.26	14.254	0.000
MVV (L/min)	82.03±19.06	84.84±21.35	1.568	0.143
ABG				
pH	7.42±0.04	7.42±0.02	0.393	0.701
PO_2_ (mmHg)	68.38±5.62	69.46±5.21	2.053	0.063
PCO_2_ (mmHg)	41.85±3.24	39.62±3.88	3.713	0.003
SaO_2_ (%)	91.15±2.08	91.46±2.03	1.298	0.219
ABG: arterial blood gas; pH: potential of hydrogen; PO_2_: partial pressure of oxygen; PCO_2_: partial pressure of carbon dioxide; SaO_2_: arterial oxygen saturation				

**3:** PPR组COPD亚组肺功能指标增加情况 The added value of pulmonary function in various degree COPD

	Mild COPD	Moderate to severe COPD	*t*	*P*
FEV_1_ added value (L)	0.06±0.05 (2.8%)	0.16±0.05 (8.6%)	6.628	0.000
FVC added value (L)	0.00±0.19 (0.0%)	0.05±0.17 (1.7%)	0.793	0.432
PEF added value (L/s)	0.81±0.23 (17.9%)	0.87±0.21 (22.6%)	0.868	0.390
MVV added value (L/min)	0.90±5.59 (1.0%)	2.80±6.20 (3.4%)	0.968	0.339

### 2.3 住院时间比较

PPR组与CS组比较，前者因术前在院行康复训练，术前住院时间[(8.30±2.22) d *vs* (4.79±1.69) d]及总住院时间[(17.23±4.18) d *vs* (14.41±4.03) d]较后者明显增加(均*P* < 0.05)，但二者术后住院时间并无显著差异，结果见表 4。

**4:** 住院时间及术后并发症情况 LOS and PPCs

	PPR group	CS group	Test value	*P*
LOS (Mean±SD)				
Total (d)	17.23±4.18	14.41±4.03	*t*=3.388	0.001
Preoperative (d)	8.30±2.22	4.79±1.69	*t*=8.941	0.000
Postoperative (d)	8.93±3.78	9.62±3.98	*t*=0.871	0.386
PPCs				
Delayed tracheal extubation	3 (7.0%)	5 (8.6%)	*χ*^2^=0.091	0.762
Pneumonia	8 (18.6%)	17 (29.3%)	*χ*^2^=1.519	0.218
Atelectasis	1 (2.3%)	1 (1.7%)	*χ*^2^=0.046	0.830
Respiratory failure	1 (2.3%)	2 (3.4%)	*χ*^2^=0.108	0.742
LOS: Length of stay; PPCs: postoperative pulmonary complications.				

### 2.4 术后并发症情况

两组患者术后肺部感染分别为：PPR组8例，发病率18.6%，CS组17例发病率29.3%，差异无统计学意义(*P* > 0.05)。两组患者术后延迟气管拔管、术后肺不张、呼吸衰竭发生率均无统计学差异(*P* > 0.05)，结果见表 4。

## 3 讨论

外科手术目前仍是早-中期非小细胞肺癌治疗的首选，术前心肺功能状态是限制患者能否手术的重要因素之一。由于人口老龄化、环境等因素的影响，肺癌合并COPD的患者势必会越来越多，COPD患者由于长期慢性缺氧，可引起患者力量下降、营养差、全身慢性炎症反应等，可导致患者围手术期并发症增加，甚至部分患者因为临界状态肺功能而不能接受手术或仅能接受姑息性手术。在术前给予这部分患者相应治疗，以期可以降低术后并发症风险，符合目前加速康复外科的核心理念。

肺康复训练是一项多学科综合干预的措施，目前已有循证医学的证据表明对于慢性呼吸系统疾病患者采取积极的肺康复训练可有效改善患者的运动耐力及生活质量^[4-7]^。其核心内容是运动训练，通过有计划的、规范的呼吸肌训练及肢体耐力训练，改善患者呼吸困难、呼吸肌无力，进而提高患者术后运动耐力，引导患者术后正确的咳嗽方式，进而降低术后患者因排痰不畅导致的肺部感染、呼吸衰竭的并发症，对于COPD患者尤其适用。常用的呼吸肌训练方法主要有三种：正常CO_2_高通气法、阻力呼吸法、闭值压力负荷训练器等。本研究中术前采取腹式呼吸、缩唇呼吸、登楼梯训练、三球式呼吸训练器等方法进行物理肺康复训练，主要目的是加强呼吸肌力量及耐力，训练患者掌握正确的咳嗽、咳痰技巧，这三种方法操作简便易行，患者依从性高，同时对于场地、专业器械等基础条件要求低，易于普及。Maria等^[8]^的随机对照试验研究发现，通过术前4周的力量及耐力训练可有效改善肺癌患者手术前FVC、最大吸气压力、最大呼吸压力、6 min步行距离(均*P* < 0.05)同时降低术后呼吸系统并发症、缩短术后住院时间、缩短胸腔闭式引流时间。Licker等^[9]^进行的随机对照研究发现，通过平均25天的术前高强度间歇康复训练，患者的峰值耗氧量及6min步行距离明显增加，与常规治疗组比较，术前康复训练可降低术后肺不张发生率及术后麻醉复苏室停留时间。但该作者也同时报道了这部分患者术前的肺康复训练并不能改善肺叶切除术后远期心肺功能^[10]^。Adrichem等^[11]^的随机单盲研究发现，术前进行吸气肌力量训练可减少患者食管癌术后肺部并发症、住院时间、再次气管插管风险。本研究的统计数据发现，经过1周的高强度肺康复训练，患者行肺叶切除总住院时间因康复训练影响有明显增加，但术后住院时间并无显著差异，这可能与总体例数偏少，结合医患关系紧张大背景下，并非所有患者达到出院标准后均立即出院、部分患者术后早期化疗、出院后继续治疗的便利等因素相关。肺康复训练组术后肺部感染发生率较常规治疗组下降(18.6% *vs* 29.3%)，虽然经检验无明显统计学差异(*P* > 0.05)，但已经出现明显下降趋势，随着统计样本量增加，可能会得到阳性结果。

Mujovic等^[12]^报道，通过术前2周-4周肺康复训练可有效改善合并COPD的非小细胞患者各项肺功能指标(FEV_1_、VC、PEF50、PEF25)及6 min步行距离，改善心肺运动耐力，对于基础肺功能越差的患者，康复训练后肺功能改善越明显。Divisi等^[13]^报道，针对27例非小细胞肺癌合并COPD患者进行4周-6周康复训练后，患者动脉血氧分压(PaO_2_)、峰值耗氧量(VO_2_)、FEV_1_均显著增加，所有患者均行肺叶切除手术，术后并发症发生率为15%。Hashmi等^[14]^研究发现，针对FEV_1_ < 1.6 L的肺癌患者进行为期8周的肺康复训练，肺康复方案包括：戒烟、心理咨询、呼吸训练、咳嗽训练、药物康复等，康复训练后患者肺功能FEV_1_、一氧化碳弥散量(DLCO)显著改善(*P* < 0.05)，但术后住院时间、ICU入住时间、并发症发生率、死亡率等无显著差异。Vagvolgyi等^[15]^研究发现，通过3周的围手术期(包括术前、术后)肺康复训练(包括戒烟、心理干预、呼吸肌训练及心肺运动训练)，均可明显改善患者FEV_1_、FVC、6min步行距离、握力、生活质量评分(CAT量表及mMRC量表)；其中术前肺康复训练可使FEV_1_、FVC、6min步行距离训练前后增加 > 10%，术后康复训练上述指标增加7%-20%。从我们的统计数据中也可发现，通过肺康复训练可改善肺癌合并COPD患者术前肺功能状况，其中FEV_1_及PEF改善明显，与文献报道相符，其中FEV_1_较康复训练前增加约4.4%，PEF较前增加约19.2%，差异均有统计学意义(*P* < 0.001)。按COPD不同的亚组分析，中-重度COPD患者康复训练后各项肺功能指标增加更明显，可能提示这类患者经肺康复训练后获益可能更大。

呼吸峰流速(peak expiratory flow, PEF)是反映气道通畅性的一个指标，是由腹肌及膈肌快速收缩产生的爆发性通气，与咳嗽动作的发生机制相同。有研究^[16]^发现PEF对于肺癌患者术后肺部并发症有预测作用，当PEF≤320 L/min时术后肺部并发症可能性大；Kera等^[17]^通过多元回归分析发现PEF值与肌肉减少症密切相关，并可作为预测呼吸肌及骨骼肌减少的敏感指标；因此PEF值在一定程度上可反映患者咳嗽排痰的能力。赖玉田等^[18]^通过前瞻性随机对照研究发现，经过短期高强度肺康复训练，可明显提高患者术前PEF值[(268.40±123.94) L/min *vs* (343.71±123.92) L/min, *P* < 0.05]。Kulnik等^[19]^的研究证明咳嗽时的呼气峰值流速与急性脑卒中患者肺部感染发生率呈负相关。

6min步行试验(6-min moving distance, 6MMD)是心肺运动试验的一种，操作简便、安全且患者耐受性好，可有效用于COPD患者心肺功能、生活质量评估。本研究中由于部分患者6MMD资料不全，未纳入统计，但目前已有越来越多研究者发现6MMD在肺癌手术患者术前评估中扮演着重要的角色，并与FEV_1_、FVC、峰值摄氧量(peak oxygen uptake, VO_2_peak)/kg等检查结果的相关性高，与肺叶切除后的手术并发症也有相关性。Irie等^[20]^回顾性分析188例胸腔镜下肺叶切除的Ⅰ期非小细胞肺癌患者，通过多元*Logistic*回归分析方法，预测合并COPD、6MMD、病理分期是患者术后肺部并发症的独立危险因素。Ginsburg^[21]^的研究发现当VO_2_peak% < 50%，且6MMD≤300 m，肺部并发症的发生风险会增加，术后出现肺部并发症患者6MWD较无并发症患者短。Marjanski等^[22]^的研究也发现当术前6MMD < 500 m时，行肺叶切除术后，肺部并发症、心房纤颤、输血的发生率明显增高，分别为60.6%、21.2%、18.1%(6MMD > 500 m时，发生率分别为36.9%、11.7%、9.0%)，平均住院日也延长至7 d(6MMD > 500 m时为6 d)。

本研究还有一些不足之处，试验设计为前瞻性非随机对照研究，而总体样本量偏小，因此无法排除多种因素所导致的选择性偏倚，这可能是一部分结果与文献报道不符的原因(如术后住院日、术后肺部感染发生率等)，这些因素需要加大样本量纳入或设计严格随机对照试验来进一步验证。但从现有的数据我们可以发现，术前短期高强度肺康复训练可改善患者心肺功能，可使一部分肺癌合并COPD患者获益，为那些因合并COPD肺功能较差的肺癌患者提供一个可靠的治疗方案。
